# Cellular and Molecular Regulatory Signals in Root Growth and Development

**DOI:** 10.3390/ijms26073426

**Published:** 2025-04-06

**Authors:** Guzel Kudoyarova

**Affiliations:** Ufa Institute of Biology, Ufa Federal Research Centre of the Russian Academy of Sciences, Pr. Octyabrya, 69, 450054 Ufa, Russia; guzel@anrb.ru

The responses of root growth and development to environmental changes ensure that plants adequately adapt to the availability of water and nutrients. For example, accelerating root elongation allows them to reach deeper soil layers, where water is still stored during drought, when the top layers become dry. Increased root branching in the soil facilitates the acquisition of mineral nutrients by plants. These types of responses are initiated by environmental signals that induce and interact with endogenous signals, resulting in changes in the division, elongation, and differentiation of root cells. Although the complicated net of these signals has been thoroughly studied, many questions still remain unanswered. The articles published in the present Special Issue are devoted to the influences of different external factors on root growth and development, as well as the molecular mechanisms involved in the control of plant growth responses.

Two articles report the involvement of microRNAs in the regulation of processes associated with the growth and development of root systems [1,2]. These small non-coding RNAs have attracted great interest in many research areas due to their ability to regulate the expression of numerous genes at the post-transcriptional level [3]. The article by Liu et al. [1] describes the results of a study on the participation of microRNAs in the morphological acclimation of sugar beet roots to nitrogen deficiency. Nitrogen is one of the most important macro-elements involved in the processes of plant growth and development, being the main component of proteins, nucleic acids, phospholipids, chlorophyll, hormones, vitamins, and alkaloids [4]. Despite intensive fertilization, plants often experience nitrogen deficiency due to the high solubility of nitrates, which are easily washed out by heavy rainfall [5]. Due to this process, nitrates penetrate deeper into the soil, and the optimization of nitrogen capture can be due to the quicker root elongation and exploitation of deep soil layers [6]. The experiments described by Lui et al. [1] were carried out on the sugar beet germplasm tolerant to low nitrogen levels [7]. They found that a low nitrate concentration in the nutrient medium led to an increase in root length and branch numbers in sugar beet after 7 d of exposure to low nitrogen compared to the control (5 mmol/L vs. 0.5 mmol/L of nitrate). The results obtained in the present study are in accordance with those of Lv et al. [8], who showed an increase in total root length and number of lateral roots in maize under mild nitrogen deficiency. A total of 22 differentially expressed microRNAs (DEMs) were identified in sugar beet root under low-nitrogen treatment. Among them, the upregulated miR156a was identified as a key DEM that potentially targets and regulates squamosa promoter-binding-like proteins (SPLs) through the microRNA–mRNA network. SPL genes encode transcription factors that play important roles in plant phase transition and plant architecture, while SPL genes are post-transcriptionally regulated by microRNA156 [9]. Yu et al. [10] demonstrated that exogenous auxin application in Arabidopsis promotes microRNA156 expression and enhances lateral root growth, confirming the involvement of microRNA156 in the control of root branching. The overexpression of the microR156a gene of *Beta vulgaris* in transgenic Arabidopsis promoted root growth, increasing the length, surface area, and volume, while its silencing had an opposite effect. The authors concluded that microRNA156 plays crucial roles in the development and acclimation of sugar beet root to low-nitrogen conditions by enabling sugar beet roots to take up nutrients.

Unlike the article of Lui et al. [2], Meng and others found a negative effect of microRNA390 on the maize root system. Its knockdown enhanced maize brace root growth. Brace roots are unique stem-borne organs developing from aerial stem nodes, which can remain aerial or grow into the ground. Those that grow into the soil have been shown to directly support corn stalks [11]. The removal of brace roots made the stalks more flimsy. Citing literature data [12,13], Meng et al. argue that brace root architecture is a critical determinant of maize’s stalk anchorage and nutrition uptake, influencing root lodging resistance, stress tolerance, and plant growth. By sequencing small RNAs from samples in the germination and growth stages, they identified key microRNAs that control the growth of maize brace roots. Brace root development was altered in inbred maize line B73 by the manipulation of microRNA390, resulting in the changes in its downstream-regulated auxin response factors (ARFs). Along with mi390, miR167, miR166, and miR172 were also found to be involved in the regulation of maize brace root growth. Using short tandem target mimic (STTM) technology, maize lines with reduced miR390 expression were obtained and their root architecture was analyzed compared to wild-type controls. It was shown that lines with reduced miR390 expression exhibit enhanced brace root length and increased whorl numbers. Gene expression analyses revealed that the suppression of miR390 leads to the upregulation of its downstream-regulated ARF genes, thereby altering root architecture. The authors consider that the results of their study provide a genetic basis for breeding maize varieties with improved lodging resistance and adaptability to diverse agricultural environments. It was shown previously that miR390 is involved in regulating root growth by influencing auxin response factors (ARFs) [14,15]. However, the specific roles of miR390 in maize brace root development remained unclear and was clarified in the present article.

A root system with a steep angle of the main and lateral roots ensures deep rooting, thereby enhancing access to water in deep soil layers, which is critical under drought, when the upper soil layers dry out [16,17]. DEEPER ROOTING 1 (DRO1), orthologs and paralogs of which have been identified in *Arabidopsis*, rice, and other species, is one of the genes involved in the regulation of the lateral root angle belonging to the IGT family [18]. In the article presented in this Special Issue, the study was carried out on *Cucumis sativus* plants [19]. The choice of this plant species was due to the peculiarity of the initiation of the lateral roots in them: unlike most higher plants, in which the formation of lateral root primordia is induced in the elongation zone of the parental root [20], in cucumber plants, it occurs within the parental root meristem [21]. In the present research, orthologs and paralogs of the DRO1 gene were identified in cucumber plants using a phylogenetic analysis of IGT protein family members. Since DRO1 expression was shown to be negatively regulated by auxin [22], the transcriptional responses of CsDRO1, CsDRO1-LIKE1 (CsDRO1L1), and CsDRO1-LIKE2 (CsDRO1L2) to exogenous auxin were analyzed and only CsDRO1L1 was found to be auxin-responsive. An analysis of transgenic plants transformed with constructs containing the promoter regions of CsDRO1, CsDRO1L1, and CsDRO1L2 fused to the H2B-mNeonGreen demonstrated an expression of the genes in the meristem in cell files of the central cylinder, endodermis, and cortex, the three genes displaying different expression patterns in cucumber roots with only partial overlap. CRISPR/Cas9 gene editing used for the knockout of individual CsDRO1, CsDRO1L1, and CsDRO1L2 genes showed that individual genes do not affect the angle of lateral root development. The authors conclude that the further development of CRISPR-Cas9-based genome editing, which allows the simultaneous knockout of multiple genes [23], should be applied in the future to simultaneously knockout two or three cucumber DRO1 homologs in order to analyze their combined role in controlling the angle of lateral roots in the stage of their emergence from the maternal root.

The theme of adaptation to nitrogen starvation is continued in another article of this Special Issue [24]. In this study, wheat plants were grown in 1/2-strength Hoagland’s solution. A nitrate concentration of five micromoles was in the control variant, while in the low-nitrate variant, it was reduced to 0.1 mM. Since growth responses to nitrogen starvation were expected to be auxin-mediated [25], 2,3,5-triiodobenzoic acid (TIBA), a known inhibitor of polar auxin transport [26], was added to half of the plants grown in the low-nitrate medium. It was found that, two days after the start of experiments, nitrogen starvation increased the number of lateral roots compared to the control, while the addition of TIBA significantly decreased their number. Transcriptomic analysis revealed the induction of gene expression related to auxin synthesis and transport and cell wall remodeling by nitrogen starvation, as well as the suppression of the effects by the inhibitor of auxin transport. Furthermore, a lowered concentration of nitrates increased the activity of enzymes involved in auxin synthesis and elevated the concentration of auxin precursor (tryptophan) and auxin IAA (indolyl acetic acid). Fourier-transform infrared spectroscopy combined with atomic microscopy revealed that the content of cell wall polysaccharides decreased, while the cell wall elasticity of the primordia of lateral roots increased under the low-nitrate conditions. The effects of nitrogen starvation on IAA synthesis and polar transport, cell wall remodeling, and lateral root development were abolished when TIBA was applied. These findings indicate that NO_3_^−^ starvation may improve auxin homeostasis and the biological properties of the cell walls of lateral root primordia, thereby promoting the initiation of lateral roots, while the inhibitor of auxin transport dampens the effects of low-nitrate concentration on auxin signaling, gene expression, physiological processes, and the root architecture. Since the stimulation of lateral root development at low-nitrogen concentrations was found not only in the present study but also in maize [27], rice [28], and other species, while larger root systems help plants increase nitrogen uptake [29], the authors recommend using significantly lower rates of nitrogen fertilizer in the seedling stage than recommended by conventional methods of fertilizer application to improve crop yields.

An article by Kuznetsova et al. [30] presents the results of the Whole-Genome Sequencing and analysis of the *Raphanus sativus* L. line capable of spontaneous tumor formation on their tap roots. Although higher plants contain functional orthologs of many mammalian tumor suppressors and oncogenes, mutations in these genes in plants have not led to tumor formation. This information suggested the existence of a completely different principle of organization of the systemic control of cell division and differentiation in plants [30,31,32]. With this argument in mind, the authors of the present paper [30] attempted to find the genes responsible for spontaneous tumor formation in the radish line by comparing its genome with the genome of plants incapable of forming such tumors. The genomes of two closely related radish inbred lines that differ in their ability to spontaneously form tumors were sequenced using Oxford Nanopore and Illumina technologies. This approach resulted in the identification of a large number of single-nucleotide variants (SNVs), which are likely to be associated with the spontaneous tumor formation. Genes that regulate the cell cycle, meristem activity, gene expression, and metabolism and signaling of phytohormones were identified among detected SNVs, and the results were validated with their Sanger sequencing. Then, the presence of these SNVs was checked in other tumor lines of the radish genetic collection, and the ethylene-responsive transcription factor ERF118 gene [33] was found to have SNVs in the majority of tumor lines. In addition, genes of the CLAVATA3/ESR (CLE) and WUSCHEL (WOX) families of radish were analyzed in this work. In plants, the CLE genes encode a large family of signaling peptides that modulate diverse developmental and physiological processes [34], while WUS genes encode homeobox transcription factors that promote stem cell maintenance [35]. In the present paper, new, previously unknown CLE genes were identified in radish. The results obtained provide a basis for investigating the mechanisms of plant tumor formation.

One more interesting aspect of root growth is addressed in [36] describing the root thigmomorphogenesis of lettuce in hydroponics. This phenomenon refers to changes in plant growth and development in response to mechanical stimuli [37]. Due to the ease of observation and study of the air environment, thigmomorphogenesis has been mainly studied based on morphological changes in the stem, leaves, and flowers [38,39,40]. In roots, thigmomorphogenesis can occur when roots experience pressure and friction from the flow of a nutrient solution in hydroponics. Despite some technical challenges, hydroponics has been widely applied in modern agriculture and has great development potential, since they provide a more stable growing environment, thus allowing plants to grow faster and produce higher yields while reducing the spread of soil-borne diseases and pests [41,42,43]. Previous studies have shown that plant growth is stimulated at nutrient flow rates in hydroponics of no more than 4 L/min [5,6]. Although higher flow rates might allow for an adequate nutrient mixture and bring more oxygen, plant growth was inhibited. The present research aimed to reveal the reason for the negative effect of the high flow rate of the nutrient solution in hydroponics. Compared with the plants without flow, the plants with a high flow rate showed a decreased root fresh weight, total root length, root surface area, and root volume but increased average root diameter and root density. The roots with flow had more upregulated metabolites than those without flow, suggesting that the flow may trigger changes in plant metabolism. The most significant enrichment was found in the arginine biosynthesis pathway. Arginine was present in all the groups and exhibited greater concentrations in the roots with flow than without it. It can be speculated from the results that a high-flowing environment of nutrient solution promotes arginine synthesis, resulting in changes in root morphology. The findings provide insights into root thigmomorphogenesis controlled by the environment and help understand how plants respond to mechanical forces.

One more paper of this Special Issue addresses the involvement of jasmonates and auxins in the inhibition of root growth under salinity [44]. Studies of plants exposed to salt stress have mainly focused on their aboveground parts [45,46]. However, recently, a number of works have appeared in which more attention has been paid to the processes occurring in plant roots under salinity [47,48,49]. Changes in the architecture of roots have been shown to play an important role in the adaptation of plants to salinity [48]. Due to the heterogeneity of the soil, salts are unevenly distributed, and the inhibition of the growth of roots that reach saline areas of the soil reduces their contact with a high concentration of soil solution. In addition, growth inhibition releases resources for the realization of protective mechanisms [46]. Jasmonates play an important role in the regulation of root growth, with the ability of these growth regulators to inhibit root elongation being one of their characteristic properties [50,51]. It has been shown that the influence of jasmonates on root growth depends on their cross-talk with auxins [52]. In the present study, an immunohistochemical approach using specific antibodies against auxins and jasmonates was used to detect the presence and levels of these hormones in root cells. The obtained data suggest the participation of either auxins or jasmonates in the inhibition of cell division, which leads to a decrease in the size of the meristem zone. The level of only auxin and not jasmonate increased in the elongation zone. However, since some literature evidence argues against the inhibition of root cell division by auxins, while jasmonates have been shown to inhibit this process, the authors came to the conclusion that elevated jasmonate is a more likely candidate for inhibiting root meristem activity under salinity conditions. The data suggest that auxins, not jasmonates, reduce the cell size in the elongation zone of salt-stressed plants, a suggestion supported by the known ability of auxins to inhibit root cell elongation.
